# First Detection of *Cryptosporidium parvum* in the Endemic Cyprus Mouflon (*Ovis gmelini ophion*)

**DOI:** 10.1007/s11686-023-00747-1

**Published:** 2023-12-08

**Authors:** Kyriacos A. Hasapis, Iris Charalambidou, Chad Schou, Catherine O’Dowd Phanis, Stefanie Kazamia, Nicolaos Kassinis, Eleftherios Hadjisterkotis, Panagiotis Karanis

**Affiliations:** 1 Department of Basic and Clinical Sciences, University of Nicosia Medical School, 21 Ilia Papakyriakou, 2414 Engomi, P.O. Box 24005, 1700 Nicosia, Cyprus; 2 Department of Life Sciences, School of Life and Health Sciences, University of Nicosia, Nicosia, Cyprus; 3 Game and Fauna Service, Ministry of Interior, 1453 Nicosia, Cyprus; 4 Agricultural Research Institute, Ministry of Agriculture, Rural Development and Environment, Nicosia, Cyprus; 5 Medical Faculty and University Hospital, University of Cologne, 50923 Cologne, Germany

**Keywords:** *Cryptosporidium parvum*, Cyprus mouflon, Free-ranging goats, Wild sheep

## Abstract

**Purpose:**

*Cryptosporidium* is an intestinal zoonotic protozoan parasite that infects domesticated and wild animals. There are no reports on the prevalence and molecular characterisation of *Cryptosporidium* in the endemic Cyprus mouflon. The mouflon is strictly protected by national and international legislation. Its main distribution is Paphos State Forest and surrounding areas, where it may share the same water sources as free-ranging domestic goats. Therefore, the present study aimed to determine the prevalence of *Cryptosporidium* spp. and genotypes in mouflon and free-ranging goats within the mouflon range.

**Methods:**

Faecal samples of 70 mouflons and 34 free-ranging goats were screened for *Cryptosporidium* by PCR amplification and sequencing.

**Results:**

Only one sample (1/70) belonging to a mouflon was PCR positive for *Cryptosporidium*. Based on sequencing of the *18S* rRNA locus, this species was identified as *Cryptosporidium parvum* (*C. parvum*). No positive sample was detected in the free-ranging goats (0/34).

**Conclusion:**

This is the first report on the molecular identification of this *Cryptosporidium* species in a Cyprus mouflon. The results indicate that the prevalence of *Cryptosporidium* in Cyprus mouflon is low.

## Introduction

*Cryptosporidium* is a zoonotic protozoan parasite that infects many hosts through the faecal-oral route, including humans and domestic and wild animals [1–3]. It is one of the leading causes of diarrhoea worldwide, second only to rotavirus [4]. Numerous studies have investigated *Cryptosporidium* infection in domestic goats and sheep. The most common species isolated are *Cryptosporidium parvum* (*C. parvum*) *C. xiaoi* and *C. ubiquitum *in goats [5–15] and *C. parvum C. xiaoi C. ubiquitum* and *C. andersoni* in sheep [5–9, 11, 13, 14, 16–20]. In some studies, *C. muris C. suis C. baileyi* and *Cryptosporidium* rat genotype II have also been isolated from goats [21–24]; *C. muris, C. bovis, C. hominis* and *C. scrofarum* have also been isolated from sheep [12, 21, 23, 24]. The prevalence of the parasite varies between countries and studies and ranges from 3.9% to 62.7% in goats and 0.9% to 31.6% in sheep. The species associated with dangerous symptoms and even mortality, especially in neonatal animals, is *C. parvum* [7].

Identification of *Cryptosporidium* oocysts in animal stool samples can be performed using microscopic examination of the samples at 1000 × magnification after staining of the oocysts using the acid-fast Ziehl–Neelsen technique [25]; however, most *Cryptosporidium* species have morphologically identical oocysts; thus, they cannot be differentiated by microscopic methods. Instead, they can be differentiated by molecular biological techniques [26, 27]. Nested PCR amplification of the 18S rRNA gene using specific primers is a more sensitive method for the identification of *Cryptosporidium* in stools compared to microscopic examination [28–30]. Nested PCR is more sensitive than the classic PCR and the PCR primers were designed to detect all *Cryptosporidium* species [20, 26, 28].

The Cyprus mouflon (*Ovis gmelini ophion*) is the largest wild terrestrial mammal on the island. It is endemic to Cyprus and classified as endangered and strictly protected [31]. Its population is estimated at 2500–3000 animals, mainly located in the Paphos State Forest and surrounding areas. Low genetic variability [32] puts the population at significant risk of an illness outbreak transmitted from sympatric livestock such as free-ranging goats that frequent the same areas and share water sources [33]. In a recent study, endoparasites were found in 97% of Cyprus mouflon faecal samples, including the lungworms *Muellerius capillaris* and *Cystocaulus ocreatus* strongylid eggs, *Eimeria* spp., *Nematodirus* spp., *Trichuris* spp., and *Moniezia* spp. [34].

The purpose of the present study was to determine the prevalence and molecular identification of *Cryptosporidium* species in the Cyprus mouflon and free-ranging domestic goats for the first time in Cyprus.

## Materials and Methods

### Study Area

Paphos State Forest is situated in the Northwest part of the Troodos Mountain range. It is a mainly coniferous forest of 600 km^2^ and constitutes the most extensive and continuous forest ecosystem in Cyprus, ranging from 200 m up to the peak of Tripylos at 1352 m [35]. The predominant vegetation is the Calabrian pine (*Pinus brutia)*. Among other trees and shrubs, the area also includes the unique endemic Cyprus cedar (*Cedrus brevifolia*), as well as the endemic golden oak (*Quercus alnifolia*), along with the occasional strawberry tree (*Arbutus andrachne*), turpentine tree (*Pistacia terebinthus*) and sumac (*Rhus coriaria*) [36]. Various flocks of livestock, mainly goats and sheep, utilise the forest and surrounding areas and mingle with the Cyprus mouflon population.

### Sample Collection

Faecal samples from 70 Cyprus mouflon and 34 free-ranging goats were collected from the ground in Paphos State Forest (Cyprus) between November 2021 and August 2022. Sampling was carried out along mouflon trails, bedding areas, and near water points, including where livestock are regularly seen. Mouflon faecal material pellets differ from goat pellets, since the mouflon is a wild sheep *versus* free-ranging goats, and were identified in this study by experienced rangers. Each fresh faecal sample (4–10 g each) was placed in a sterile polystyrene tube (50-ml centrifuge tube) with records of the date, location, and identification number. The samples were transferred to the laboratory on the same day and were stored at 4 °C until DNA extraction within 3 weeks after collection.

### DNA Extraction and PCR

The total genomic DNA of each faecal sample was extracted using a QIAamp Fast DNA Stool Mini Kit (Qiagen, Hilden, Germany) according to the manufacturer’s instructions. *Cryptosporidium* species were determined by nested Polymerase Chain Reaction (PCR) amplification of an *18S* rRNA locus fragment (600 bp) using previously described primers [20]. The primers used for the first amplification were SHP1 (forward) 5ʹACC TAT CAG CTT TAG ACG GTA GGG TAT 3ʹ and SHP2 (reverse) 5ʹ TTC TCA TAA GGT GCT GAA GGA GTA AGG 3ʹ. The primers used for the second amplification were SHP3 (forward) 5ʹ ACA GGG AGG TAG TGA CAA GAA ATA ACA 3ʹ and SSU-R3 (reverse) 5ʹ AAG GAG TAA GGA ACA ACC TCC A 3ʹ. The cycling conditions used in both amplifications were 94 °C for 3 min, 35 cycles of 94 °C for 45 s, 56 °C for 45 s and 72 °C for 60 s, followed by a final extension of 72 °C for 7 min.

### Sequencing and Phylogenetic Analysis

The DNA band of the positive PCR product was extracted from an agarose gel and purified using the Blirt ExtractMe DNA Kit (Blirt, Gdansk, Poland). The purified PCR product was sent for sequencing (using the forward primer of the nested PCR reaction) to Macrogen Ltd Europe, Amsterdam. For the determination of *Cryptosporidium* species, the sequence was subjected to BLAST (https://blast.ncbi.nlm.nih.gov/Blast) searches at NCBI GenBank. The sequence was deposited in the NCBI GenBank under the accession number: OQ287831. Using the Neighbour-Joining method in MEGA 11 software, a phylogenetic tree was constructed with Bootstrap 1000 replicates. Evolutionary distances were calculated using the Kimura two-parameter model (https://www.megasoftware.net/) [37].

## Results

Only one Cyprus mouflon sample (1/70 = 1.4%) was PCR positive for *Cryptosporidium* and was further identified by DNA sequencing and Blastn analysis as *C. parvum*. No positive sample was detected in the free-ranging goats (0/34).

**Fig. 1:**
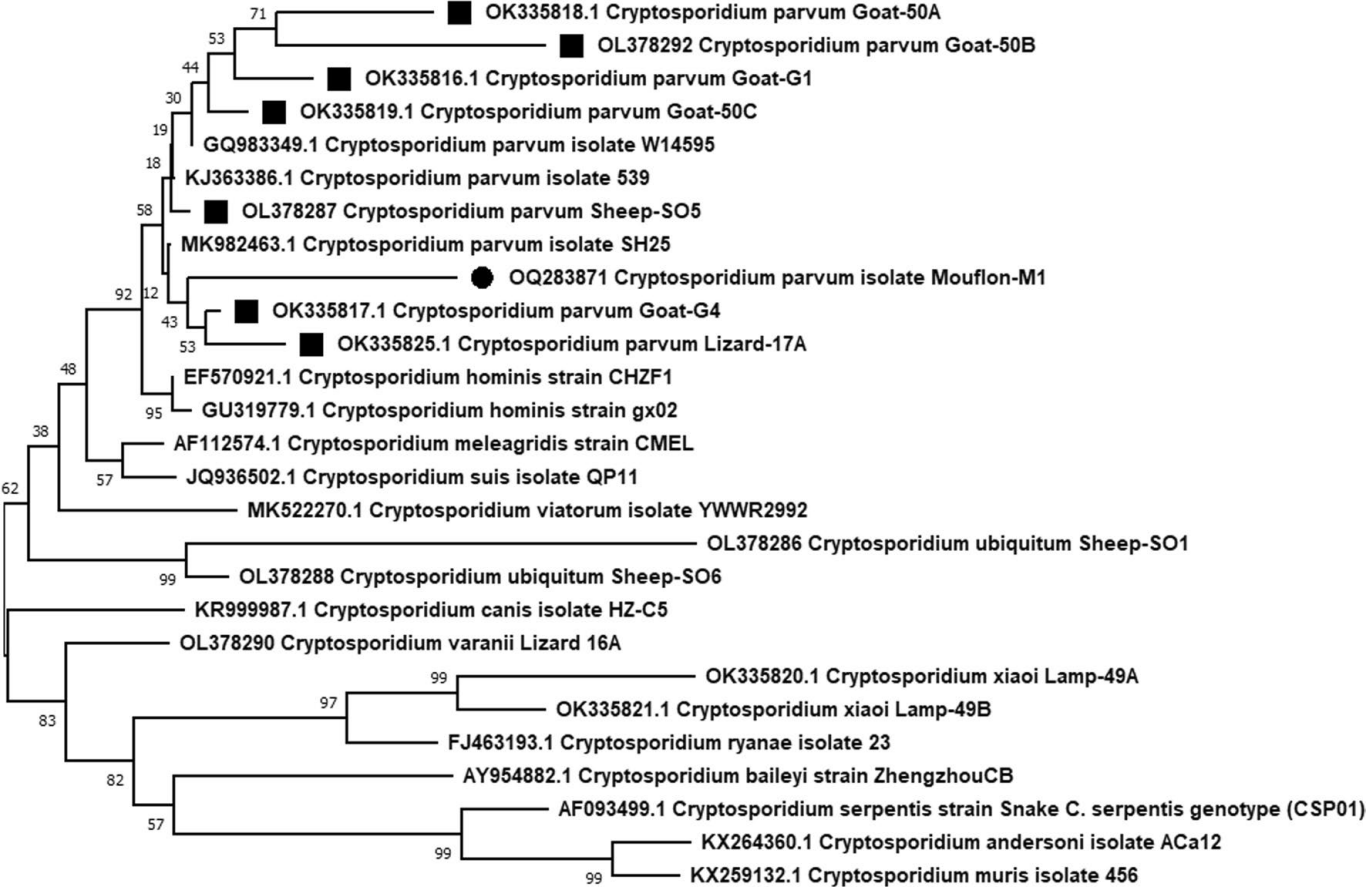
Phylogenetic analysis of *Cryptosporidium* species based on the 600 bp sequence of the 18S rRNA gene, using the Neighbour-Joining method with Bootstrap 1000 replicates. Evolutionary distances were calculated using the Kimura two-parameter model. The percentage of bootstrap samplings is indicated by the numbers above the branches. The phylogenetic tree was constructed using the MEGA 11.0 software. The *Cryptosporidium* species isolated from a Cyprus mouflon (*Ovis* *gmelini ophion)* in this study is marked with a solid circle. All the other sequences are reference sequences deposited in Genbank. The *Cryptosporidium parvum* sequences that were analysed from animals isolated in Cyprus (Schou *et al*. [5]) are marked with a solid square

The phylogenetic analysis using the Mega11 software indicated that the *18S* rRNA sequence of the *C.* *parvum* isolated in this study (solid circle in Fig. 1) formed a well-defined cluster with the respective *C. parvum* reference sequences, regardless of whether these sequences were isolated from animals in Cyprus (solid squares in Fig. 1) or from other countries (Fig. 1).

## Discussion

The present study is the first to identify *Cryptosporidium* species in the endemic Cyprus mouflon. Two other *Cryptosporidium* species, *C. ubiquitum* (previously *Cryptosporidium* cervine genotype) and *C. muris* have been found in the European mouflon (*Ovis gmelini musimon*) in the Czech Republic [38, 39]. The presence of *C. parvum* in the current study was low (1.4%), similar to *C. muris* (1/43 = 2.3%) found in faecal samples of wild European mouflon [39].

Due to the high genetic relationship between mouflon and sheep, *C. parvum* may cause severe symptoms in neonatal and young mouflon. In sheep, young infected animals are more likely to have clinical symptoms, while most of the infected adult animals are asymptomatic but still shed oocysts of the parasite [40]. The clinical signs of cryptosporidiosis include watery diarrhoea, dehydration, significant weight loss, reduced growth rate and sometimes even death. Most symptoms usually last 1–2 weeks after infection [40]. In a wild population of the Sardinian mouflon (*Ovis gmelini musimon* × *Ovis* sp.), reproductive females and young mouflon in poor body condition were found to be the main spreaders of gastrointestinal parasites [41]. Therefore, the prevalence of *C. parvum* in relation to age and gender merits further study in the Cyprus mouflon and will provide vital information for its conservation management.

Data on the epidemiology of *Cryptosporidium* in Cyprus is limited. Hoque *et al*. [42] reported high occurrence of zoonotic subtypes of *C. parvum* in Cypriot dairy farms, almost 40% of the cattle at local farms were positive for *Cryptosporidium.* The species identified were *C. bovis*, *C. ryanae* and *C. parvum*. Similarly, Schou *et al.* [5] reported a high occurrence of *Cryptosporidium* in domestic sheep (9/32 = 28%) and goats (5/10 = 50%) at local farms, although the number of samples investigated was low. The species identified were *C. xiaoi* and *C. ubiquitum* in sheep and *C. parvum* in goats. In comparison, none of the goats in the present study was infected. This may be a reflection of the different conditions faced by farmed livestock [5] compared to free-ranging animals. In a systematic review and meta-analysis of *Cryptosporidium* infections in domestic and wild ungulates, Hatam-Nahavandi *et al*. [43] found *Cryptosporidium* prevalence to be lower in wild compared to farmed populations of the same host species. Animal density and confinement to the same (contaminated) environment facilitate *Cryptosporidium* transmission in farmed animals. The outcomes of the present study support this assumption, with 1.4% *C. parvum* prevalence in the Cyprus mouflon and no positive samples in the free-ranging goats. Nevertheless, the investigated number of goat samples in the current study was low (*n* = 32) and research comparing farmed and free-ranging livestock at Paphos State Forest merits further attention.

Furthermore, infection between domestic and wild populations of closely related animal species is a common phenomenon [43]. Therefore, *Cryptosporidium* may be transmitted from goats and sheep to mouflon, and vice versa. Importantly, the only *Cryptosporidium* species found in goats and the Cyprus mouflon is *C. parvum*. The transmission of cryptosporidiosis could occur through the faecal-oral route and the contamination of shared water sources at Paphos State Forest and surrounding areas. More research is required to investigate this relationship. Sampling should focus primarily on areas where mouflon and domestic ungulates coexist, especially near artificial watering points constructed and maintained by the Game and Fauna Service in the driest parts of the mouflon range. Disease transmission risk within and between species has been identified as a factor to consider in such watering areas [44–46]. Considering that *C. parvum* is the most dangerous *Cryptosporidium* species in animals and responsible for mortality in neonatal animals, actions should be taken to protect the Cyprus mouflon considering that it is an endemic and endangered species.

## Conclusions

The results indicate a low prevalence of *Cryptosporidium* in the Cyprus mouflon and free-ranging goats. This is the first report of *C. parvum* in the endemic Cyprus mouflon. Further studies should identify the factors that influence parasite transmission in the Cyprus mouflon and the livestock it comes in contact with.

## Data Availability

The material obtained in this study is stored at the Laboratory of the Department of Life Sciences at the University of Nicosia. Representative nucleotide sequences obtained in this study were submitted to GenBank^®^ under the accession number: OQ283871.
